# Breaking an Epigenetic Chromatin Switch: Curious Features of Hysteresis in *Saccharomyces cerevisiae* Telomeric Silencing

**DOI:** 10.1371/journal.pone.0113516

**Published:** 2014-12-23

**Authors:** Vijayalakshmi H. Nagaraj, Swagatam Mukhopadhyay, Adel Dayarian, Anirvan M. Sengupta

**Affiliations:** 1 BioMaPS Institute, Rutgers University, Piscataway, NJ, United States of America; 2 Cold Spring Harbor Laboratory, Cold Spring Harbor, NY, United States of America; 3 Kavli Institute for Theoretical Physics, University of California Santa Barbara, Santa Barbara, CA, United States of America; 4 Department of Physics and Astronomy, Rutgers University, Piscataway, NJ, United States of America; Tata Institute of Fundamental Research, India

## Abstract

In addition to gene network switches, local epigenetic modifications to DNA and histones play an important role in all-or-none cellular decision-making. Here, we study the dynamical design of a well-characterized epigenetic chromatin switch: the yeast SIR system, in order to understand the origin of the stability of epigenetic states. We study hysteresis in this system by perturbing it with a histone deacetylase inhibitor. We find that SIR silencing has many characteristics of a non-linear bistable system, as observed in conventional genetic switches, which are based on activities of a few promoters affecting each other through the abundance of their gene products. Quite remarkably, our experiments in yeast telomeric silencing show a very distinctive pattern when it comes to the transition from bistability to monostability. In particular, the loss of the stable silenced state, upon increasing the inhibitor concentration, does not seem to show the expected saddle node behavior, instead looking like a supercritical pitchfork bifurcation. In other words, the ‘off’ state merges with the ‘on’ state at a threshold concentration leading to a single state, as opposed to the two states remaining distinct up to the threshold and exhibiting a discontinuous jump from the ‘off’ to the ‘on’ state. We argue that this is an inevitable consequence of silenced and active regions coexisting with dynamic domain boundaries. The experimental observations in our study therefore have broad implications for the understanding of chromatin silencing in yeast and beyond.

## Introduction

All-or-none decisions are important in many biological processes including cell-fate decisions. The choice of cell fate initially depends on particular differentiating signals, but the cell often maintains the resulting fate reliably even as the original signals disappear. These fates are often associated with gene expression patterns that are heritable, a key aspect of epigenetic phenomena. Mechanisms of such heritability range from genetic networks involving transcription factors to particular modifications of DNA or histones.

Histone tails and their possible modifications have been identified to be a critical player in cellular memory [1]. Coordination between histone modifying enzymes and other proteins that bind to modified tails seem to play a key role in that process [2]. In budding yeast, the Silenced Information Regulator (SIR) proteins control repression of gene expression from hidden mating loci and from telomeres. The enzyme playing a key role in SIR silencing is Sir2p, which deacetylates H4K16-Ac. These deacetylated sites allow binding of the Sir3p/Sir4p complex, which, in turn, leads to recruitment of more Sir2p [3].

Hidden mating loci, like HML and HMR have special sequences to which several proteins bind, some of which are crucial for recruiting Sir2p [3]. These sequence elements, known as silencers, are believed to be nucleation centers for local silencing. In wild type budding yeast, the hidden mating loci are always silenced. Silencing in these loci become epigenetic in a particular mutant, *sir1*, which purportedly weakens the ability of the silencers to recruit Sir2p [4]. This mutant system has lead to many insights into epigenetic silencing. In addition, telomeric silencing in wild-type shows epigenetic variegation [5]. This is the system that will be used in our studies.

Explaining the reason for the stability of such a state, or more precisely, the slow time scale of switching between distinct expression patterns, is essential to the systems biology of epigenetic “memory”. The dominant explanation of long-term memory in molecular biology often involves invoking high fidelity of molecular processes. It is useful to look at two examples in this regard. The first one involves genetic “memory”, and the second concerns epigenetics of DNA methylation.

What controls the stability of genetic information? A major mode of loss of genetic information is base substitution error [6]. Naively, replication error rate would be controlled by the free energy cost of improper base pairing, leading to an error rate of 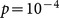 per base per replication. This is indeed the case for several DNA polymerases without proofreading mechanisms [6]. Kinetic proofreading allows the rate to go down to 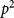 or higher powers of 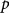 [7], [8] explaining the observed higher fidelity (10^−7^–10^−8^ per base per replication [9]) of polymerases with backtracking and exonuclease activity. Nevertheless, the error rate remains a simple function of the rate of mis-incorporation by the molecular process of replication. In other words, the fidelity of the base pair matching directly determines the timescale of genetic memory.

Our second example involves the textbook model of epigenetics of DNA methylation. This simple model posits that the stability of the methylated state is achieved by the efficiency with which the maintenance methylase recognizes and methylates hemimethylated CpG sites [10]. In addition, the rate of *de novo* methylation has to be low in order to protect the unmethylated state of CpG’s. However, the contrast between relatively high rates of error in these biochemical processes (5–15%) and the very high fidelity of the observed epigenetic states [11], [12] suggests that the simple model misses the real cause of epigenetic stability.

The model of SIR silencing holds that Sir2p, bound to a nucleosome, can act on other nucleosomes in its neighborhood, allowing silencing to spread from silencers [2]. This interaction with neighboring sites may produce interesting collective aspects of epigenetic states whereby the stability of the silenced state is caused by the ‘error correcting’ nature of the interactions with neighboring nucleosome tails and their states. With this picture of SIR silencing, it is tempting to assume that the SIR system indeed exhibits non-linear bistable behavior, explaining the robustness of epigenetic states. However, the presence of positive feedback and of relatively stable epigenetic states do not necessarily imply nonlinear bistability. A case in point: recent experimental studies of the HIV-1 Tat system showed that the phenotypic bifurcation in gene expression is most likely explained by the slow on and off rates of the Tat occupancy of the Tat promoter, rather than by the presence of positive feedback loop in the circuits [13]. A similar study in a yeast system also shows stochastic multimodal expression, while the deterministic nonlinear description of the system lacks bistability [14]. Apart from that, quantitative modeling of the SIR system shows that the simplest model is not bistable [15] and requires either greater nonlinearity [15]–[17] or additional chromatin modification states [16], [17].

We should also mention that there are several other putative mechanisms, distinct from the so-called ‘railroad’ model reviewed in Grewal and Moazed [2], proposed to explain epigenetic silencing [18]. Many of these mechanisms do not explicitly involve bistability. In summary, there are strong reasons to test experimentally whether epigenetic chromatin silencing functions like a typical bistable system, which is the tacit assumption in all the relevant computational work [15]–[17], [19].

From the lambda switch [20]–[22] to the synthetic toggle switch [23], as well as the lac operon in the presence of TMG [24], [25], the phenomenon of nonlinear bistable genetic switches has received much discussion in the systems biology literature. One defining characteristic of nonlinear deterministic bistable systems is hysteresis. This paradigm was bolstered for the lac operon by a landmark experimental investigation of hysteresis effects and the associated bifurcation diagram [25]. Therefore, it is natural that we choose a similar strategy to analyze our system.

**Figure 1:**
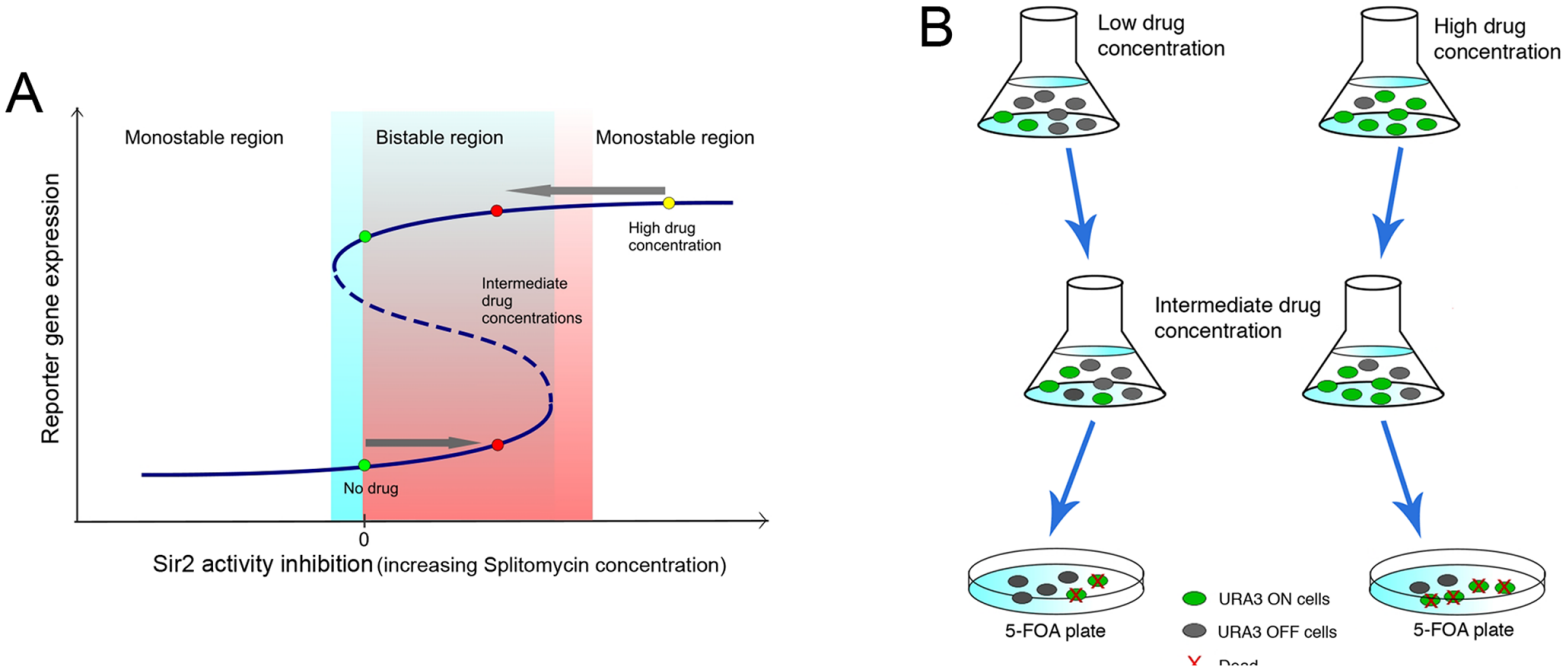
The hysteresis effect and the experimental set up. (A) In a bistable system, the system can be in two different states even for the same parameters (e.g. Sir2 activity level in the SIR silencing system). This state depends not only on the current parameters, but also on the value of the parameters in the past. (B) Two populations of yeast, which carry *URA3* gene in of the telomeric regions, are grown at two different concentrations of anti-silencing drug. One is grown for several generations in high concentration of the drug, whereas the other one is not exposed to any drug. Subsequently, the two populations are exposed to the same intermediate concentration of drug. The state of the silencing in each population can be monitored using the fact that *URA3* expressing cells die in the presence of 5-FOA.

The expectations for the signature of non-linear bistable dynamics in a traditional hysteresis experiment are based on the following assumptions. Altering a parameter (e.g. concentration of a drug) is a one-dimensional trajectory in the bifurcation diagram of the system. The generic trajectory would go from the bistable region to the monstable region via a saddle-node bifurcation [26]. This is what one expects without any fine-tuning. Theoretically, the saddle node is associated with a stable fixed point and an unstable fixed point colliding and annihilating each other. In practice, if the system was stuck to that ill-fated stable state, it has to jump to some other dynamical attractor (usually another stable state), as the control parameter moves past the saddle-node bifurcation threshold (Fig. 1A). Thus, one observes discontinuous changes in dynamical variables in response to the continuous parameter changes at the saddle-node bifurcation. In the context of systems biology, quantitative measurements of these jumps, in addition to the history dependence, give credence to the validity (or lack thereof) of describing a gene expression switch as a nonlinear bistable system [13], [25].

Theoretical studies [15], [27] indicate that the ability of chromatin silencing domains to expand in size under perturbations and the resulting titration effects alter how the system behaves as it transitions from bistable to monostable region. In other words, under some conditions, the ‘off’ state merges with the ‘on’ state, rather than exhibiting a discontinuous jump [27]. As we mentioned before, the generic finite dimensional nonlinear system goes through jump, without any fine-tuning. However, the presence of an additional condition, namely, the condition for dynamically stable boundary between silenced and active regions (akin to conditions for phase equilibrium in thermodynamics [28]), provides the extra tuning [15], [27] and could make the system go through a supercritical pitchfork bifurcation [26]. A careful experimental investigation of this transition would therefore illuminate key distinctive features of epigenetic chromatin silencing in contrast to those of regular genetic switches.

## Results and Discussion

To study hysteresis, we need one parameter with which we could perturb the system. Prime candidates for such perturbation are drugs that alter the activity of Sir2p [29]. Two of the drugs that have often been used for this purpose are nicotinamide (NAM) [30] and splitomycin (alt. splitomicin) [31]. Using such inhibitors, we can scan up and down the Sir2p activity and monitor gene expression in a population of cells for telltale signs of hysteresis (see Fig. 1A). Note that Sir2p inhibition affects the SIR silencing everywhere in the genome, as opposed to perturbations influencing transcriptional regulation at an individual promoter [19]. Inhibition of Sir2p activity by NAM has been used to study chromosomal position effects on transcriptional burst size [32] without making explicit connection to epigenetic chromatin states.

**Figure 2:**
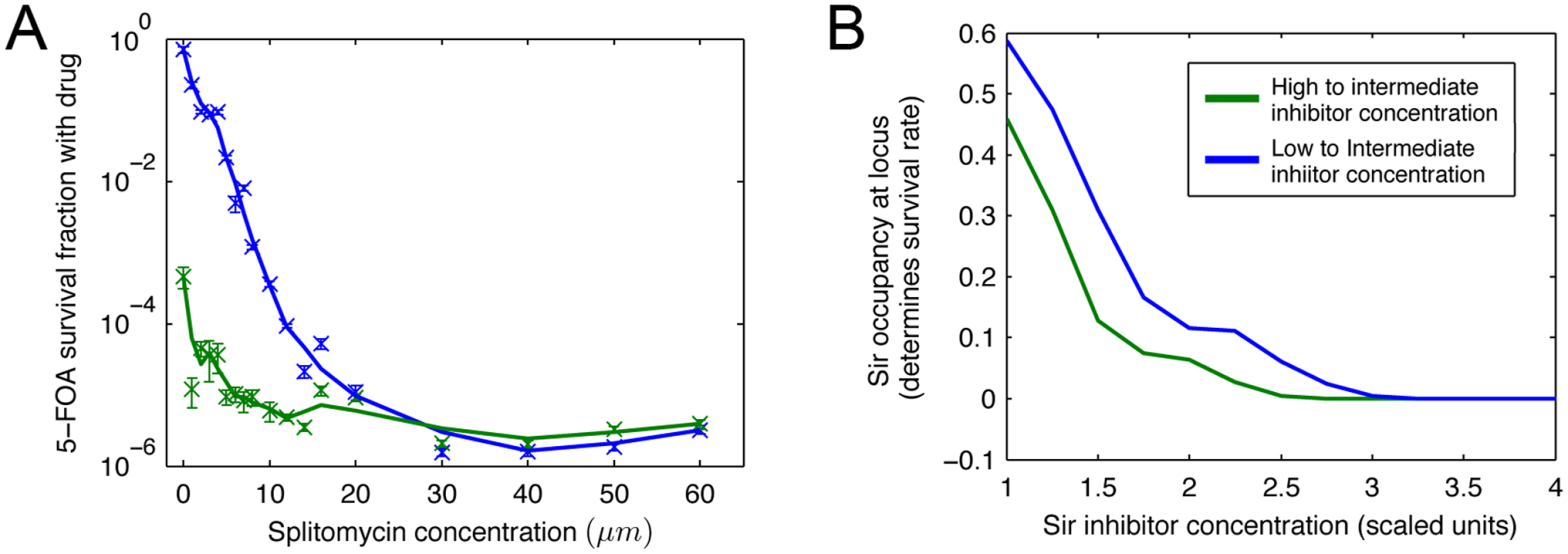
Survival rate for various intermediate concentrations of anti-silencing drugs. (A) The experimental results shown for two populations, one, which had been initially exposed to a high drug concentration (blue), and the other one which had not been exposed to any drug (green). The survival rate between the two populations differs by a few orders of magnitude for lower intermediate concentrations, consistent with bistable behavior of the wild-type. In addition, the lines for the two populations ‘meet’ past a critical concentration of about 25 µM of splitomycin, indicating a transition to a monostable ‘on’ regime. (B) The result of simulations for a model of SIR silencing.

Usually, one observes a reporter of gene expression to monitor the epigenetic state. One has many choices in this regard, ranging from classic 5-FOA [33] or *ADE* pigmentation assays [34] to measurement of single-cell fluorescent protein levels [35]. One might imagine the measurement of in vivo single cell fluorescence is the best option in this regard. In practice, instantaneous protein level is a rather noisy reporter of the underlying state of epigenetic silencing. Particular complication arises from the fact that, in the active state, transcription could be happening in intermittent bursts [36]–[39]. These bursts could happen over time scales shorter than SIR silencing switching times, creating a multimodal or broad expression profile even when the region is not silenced. On top of that, the protein expression depends on the abundance of ribosomes and correlates strongly with the size of the cell (see S1 File) making the analysis even more complicated. As a result, one rarely gets clean sharp well-separated peaks of expression in epigenetically silenced system, as seen in Fig. 2A, *sir1* data in Xu et al. [40], or in our own results, presented in Figure 1 in S1 File, based on a strain used in Bitterman et al. [30].

What is needed is a reporter that could ‘average out’ these shorter time fluctuations, providing a more reliable indication of the epigenetic state. The two classic assays mentioned before, precisely meets that criterion. Cell growth and division in the case of t 5-FOA selection and the red pigmentation in the *ADE2* assay are results of integrating expression of particular genes over several generations. These experiments track changes over days rather than over hours. Even if the relationship of the observed quantities and gene expression is non-linear, these measurements are much more reliable, compared to single-cell expression distribution, when it comes to investigating the nature of bifurcations.

We use a particular strain of yeast with *URA3* integrated at one of the telomeric silenced regions [41]. One can assay the state of silencing, since *URA3* expressing cells fail to grow in the presence of 5-FOA [33]. Although, in recent past, some doubts have been raised about appropriateness of the 5-FOA sensitivity for studying silencing [42], subsequent work [43], [44] reaffirm the ability of the assay to report telomeric position-effect variegation, namely ‘on’/‘off’ states of the *URA3* gene placed near telomere. In order to study hysteresis, we grow two different populations of yeast from the same strain, as illustrated in Fig. 1B. One population is grown in absence of any splitomycin and the other in the presence of high level of the drug. Because of the anti-silencing influence of splitomycin, the second population should have a much higher abundance of *URA3* ‘on’ cells compared to the first one. Subsequently, each of these two populations is subjected to the same intermediate concentration of splitomycin for 12–15 generations. Notice that, in that many generations, original Ura3p molecules would be diluted by a factor of 10^4^ or more. These cells are then plated both on 5-FOA plates and on YPD plates [45]. Estimated survival rates therefore inform us of the ratio of ‘on’ to ‘off’ cells at various splitomycin concentrations. If, for a particular concentration, the system is bistable, one expects very different 5-FOA survival rates for the two populations. On the other hand, if the system is monostable for that concentration, the memory of the initial state would be lost quickly over a few generations and the two survival rates will approximately be the same. If this is a genuinely non-linear switch, we expect a sharp transition from bistability to monostability as we increase the splitomycin concentration.

Fig. 2A shows the results of our 5-FOA experiments. The green line corresponds to the survival rate of the cells, which have been initially exposed to high levels of the drug. These survival rates are typically lower than the blue line, representing survival rate of the cells that did not have any initial exposure to the drug. This difference is very pronounced for lower concentrations of splitomycin, consistent with bistable behavior. More crucially, these two lines ‘meet’ past a critical concentration of about 25 µM of splitomycin. The apparent survival rate in this region of high drug level is around 10^−6^. We find that these survivors, in fact, are mutants that could thrive even in the presence of 5-FOA. Effectively, all non-mutant cells express the *URA3* gene in this regime and therefore die when exposed to 5-FOA.

**Figure 3:**
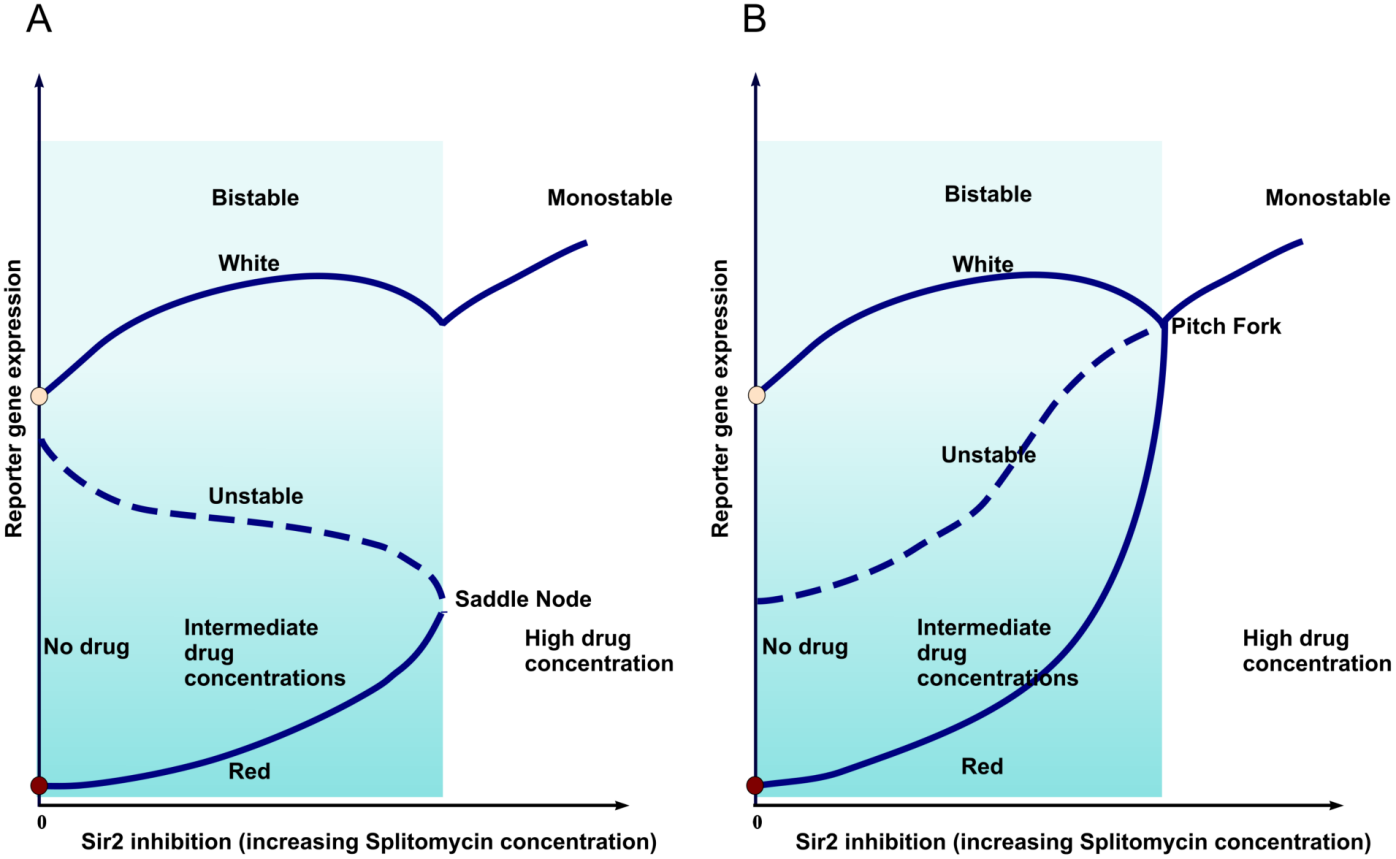
Two different possible hysteresis loops for the silencing level as a function of Sir2 activity. (A) This curve represents the gene expression level as one varies the Sir2 activity and the resulting change in the silencing level. As the system enters the monostable region with high gene expression, one of the two silencing solutions disappears and only one solution remains. In some parameter regime, there is a discontinuity between the two solutions at the transition point. Unlike in conventional saddle-node bifurcation, the slope of the upper branch is not smooth at the point of bifurcation. This is a consequence of the constraint on the total abundance SIR proteins in the cell. See Dayarian and Sengupta for more details [27]. (B) In some other parameter regime, as the system enters the monostable region with high gene expression, the two solutions merge in a continuous manner and form the single monostable solution [27].

Having established the existence of hysteresis in the system, we now come to the question of the nature of the transition to the monostable region. Previous theoretical work [27] indicates that the system can be in two different regimes, as represented in Fig. 3. In one regime (Fig. 3A), the silenced and the active regions remain very distinct but the silenced regions get eliminated as the inhibitor concentration is increased. In the other (Fig. 3B), the two states merge at a threshold concentration of the inhibitor, and give rise to a monostable intermediate state (supercritical pitchfork bifurcation). We should note that the apparent meeting of survival rates at the threshold, in the 5-FOA experiment does not indicate the merger of the ‘on’ and the ‘off’ phenotype, it means that there is just enough gene expression to turn the effective survival/growth rate effectively to zero (after discounting mutations). In order to study the nature of the monostability-bistability transition, we have to turn to a different assay, allowing us finer monitoring of the expression level.

**Figure 4:**
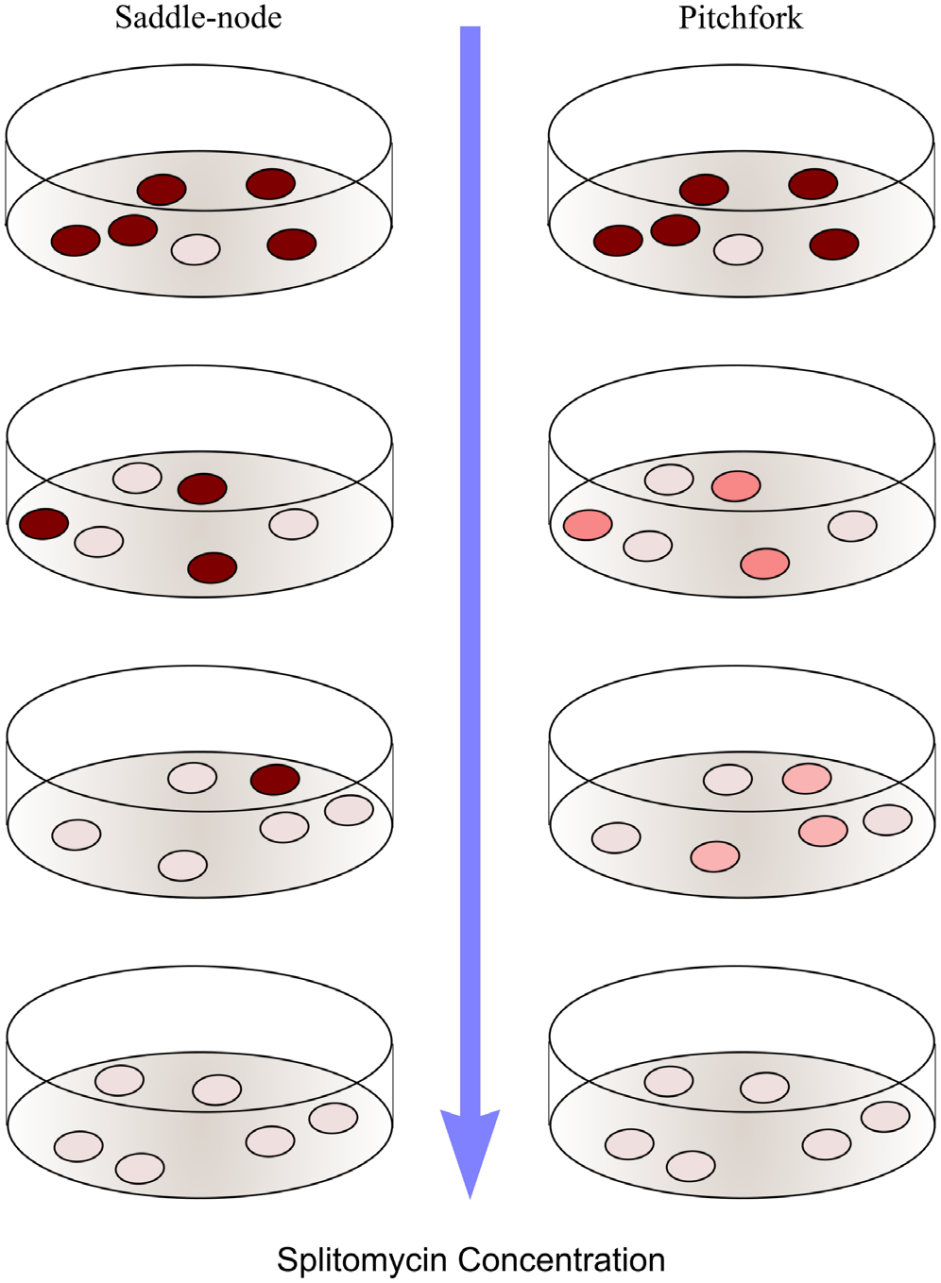
Schematic presentation of the two scenarios for the result of the *ADE2* expression experiment. Monitoring silencing of the *ADE2* gene**.** The red to dark pink colonies represent the ‘off’ state (*ADE2* gene turned off). The white to light pink color represent the ‘on’ state (*ADE2* gene is turned on). As we increase the concentration of splitomycin, the left part of the image shows the common saddle-node scenario. The pigmentation level of ‘on’ and ‘off’ states can be distinguished all the way to the threshold but their distribution keeps changing so that, past the threshold, there are more ‘off’ (deep red) states. The alternative supercritical pitchfork scenario, represented on the right, indicates a merger of pigmentation levels.

To achieve this goal, we use the colony color assay indicating *ADE2* expression level near the telomere [5]. The red to dark pink colonies represent the ‘off’ state (*ADE2* gene turned off). The white to light pink color represent the ‘on’ state (*ADE2* gene is turned on). As we increase the concentration of splitomycin, the common saddle-node scenario leads to the prediction that the pigmentation level of ‘on’ and ‘off’ states can be distinguished all the way to the threshold but their distribution keeps changing so that, past the threshold, there would be no more ‘off’ (deep red) states. The alternative, the supercritical pitchfork scenario, predicts a merger of the pigmentation levels. These two alternatives are schematically represented in Fig. 4.

**Figure 5:**
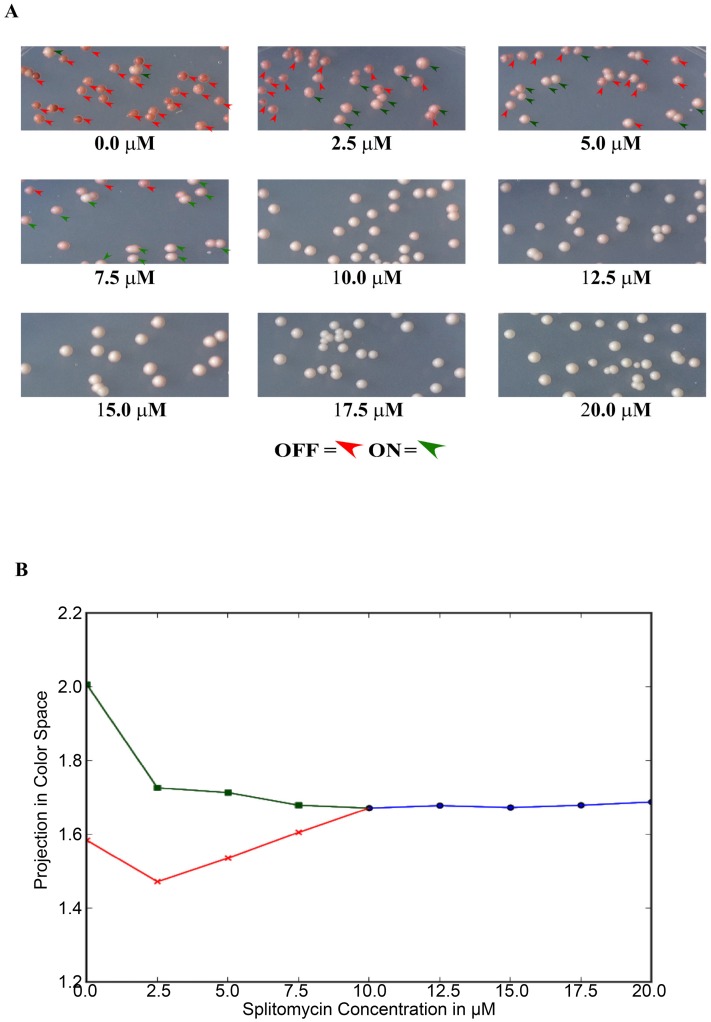
Results of the ADE2 expression experiments. (A) Images show the colonies as they grow on the plates with various concentrations of splitomycin. The red and green triangles indicate examples of ‘off’ and ‘on’ colonies, respectively, as decided by the mixture model. At 0, 2.5 and 5.0 µM splitomycin, visual classification matches the results from the mixture model perfectly, with small fraction of mismatches appearing at 7.5 µM. From 10.0 µM on we do not try classification, since, in this range, it becomes harder to make the distinction. Note that, as one increases splitomycin concentration from zero, the so-called ‘on’ and ‘off’ states come closer to each other, differing only in shades of pink, finally merging with each other into an single state at a threshold. (B) Quantitative representation of the pitchfork-like bifurcation. The plot shows a projection of the three dimensional channel intensity vectors (normalized strengths of colors red, green and blue), against the drug concentration. Average measurements of colonies corresponding to ‘off’ state (red line) and ‘on’ state (green line) merge around the concentration of 10 µM of splitomycin, and continue as the single monostable state (blue line).

Fig. 5 summarizes the results from the experiment monitoring the pigmentation levels of yeast colonies under different concentrations of splitomycin. Panel A of the figure shows the colonies as they grow on the plates with various concentrations of splitomycin. As we can see the cells grown at 0, 2.5 and 5.0 µM splitomycin show the cells in two distinct states. As we go up in splitomycin concentration, past 7.5 to 10.0 µM range it becomes harder to make these distinction. As one increases splitomycin concentration from zero, the so-called ‘on’ and ‘off’ states come closer to each other, differing only in shades of pink, finally merging with each other into a single state at a threshold. Topologically, such a merger is a characteristic of a supercritical pitchfork bifurcation [26].

The second panel of the figure is a quantitative representation of the same phenomenon. The plot shows a projection of the three dimensional channel intensity vectors (normalized strengths of colors red, green and blue), against the drug concentration. Average measurements of colonies corresponding to ‘off’ state (red line) and ‘on’ state (green line) merge around a threshold concentration (around 10 µM) of splitomycin, and continue as the single monostable state (blue line).

In summary, there is a threshold of drug concentration, past which most cells are derepressed. Even more crucially, as one approaches that threshold, the survival fraction in the 5-FOA experiment drops by five orders of magnitude or more, while splitomycin concentration increases barely by an order of magnitude. These observations are consistent with a transition from a bistable region to a monostable ‘on’ region with increasing level of the Sir2p inhibitor. Further *ADE2* pigmentation assays (associated with another telomeric locus) suggests that the ‘on’ and the ‘off’ state merge at the transition concentration.

In order to illustrate the typical outcome of a nonlinear bistable model of epigenetic chromatin silencing, we show the results of simulations of a particular model of SIR silencing [46] in Fig. 2B. The computational results indicate a transition qualitatively similar to the one observed in our experiments. Since hysteresis is a robust property of nonlinear bistable models, it is not particularly sensitive to our specific choice of model details. In the next section, we will discuss the ramifications of these results.

## Conclusions

Our observations, in conjunction with well-established results from other groups, pose strong constraints on competing proposals for mechanistic models of SIR silencing. In order to further the discussion, we need to organize these proposals into broad classes and extract qualitative predictions that are insensitive to our ignorance of many details of individual models. We will see that all viable models need a high degree of supra-molecular cooperativity, in order to be consistent with our data, especially the feature of a sharp drop in survival fraction and the appearance of a threshold. In addition, the observed pitchfork-like bifurcation structure suggests features not naturally present in all of these models.

While contrasting different mechanistic models, we acknowledge that several of these mechanisms could be present together in our system. However, for the sake of clarity, we would like to consider the extreme cases where one of these mechanisms is the key driver of silencing, and not just an additional reinforcement. It is possible that epigenetic silencing absolutely requires several of these mechanisms to be operative together. However, one should consider such complex mechanisms only when all the simpler explanations have failed.

**Figure 6:**
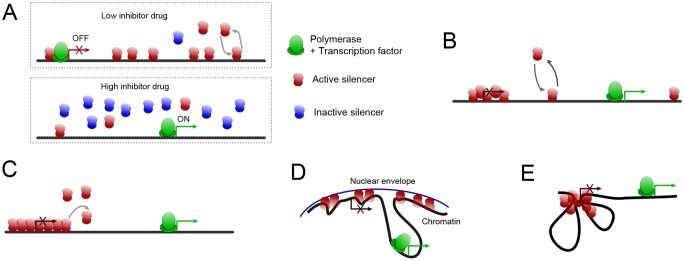
Schematic presentation of various scenarios for the SIR silencing system. (A) A highly cooperative binding scheme for the inhibitor drug to Sir2p can cause the activity of the later to change drastically as a result of small change in the drug concentration. (B) The state of each nucleosome is independent, but the transcriptional readout depends on the histone modification status of multiple (neighboring) nucleosomes. (C) Polymerization/Oozing/Railroad model: the feedback from the neighboring silenced regions leads to the spreading of silenced domain. (D) Silencing is a consequence of subnuclear localization of loci and the presence of a higher concentration of Sir proteins in the subnuclear region. (E) Thanks to DNA looping, the strong positive feedback can come from many nuleosomes being in close proximity.

Here are the models we consider, some of which are discussed further in the review by Talbert and Henikoff [18]:

A) Single-molecule cooperativity (Fig. 6A).

Perhaps the easiest way to explain such a sharp drop in the survival rate is to postulate a molecular switch mechanism, for example, a highly cooperative binding scheme for the inhibitor to its target protein Sir2p. This straw man of a model would require the fraction of active Sir2 to drop with splitomycin concentration as 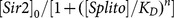 with 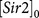 being the concentration of active Sir2 at no drug and 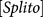 being the concentration of splitomycin. The Hill coefficient, 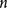, needs to be of the order of 5–6, and the dissociation constant, 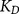, needs to be at most a few micromolars, in order to explain the observations. If these were true, *in vitro* measurements of activity of Sir2p for different splitomycin concentrations would show similar Hill coefficient and, possibly, a similar dissociation constant. The in vitro measurements of Bedalov et al. [47] would indicate the dissociation constant (if we identify it to be the IC_50_) to be around 60 µM. In addition the drop of Sir2p activity in those measurements is consistent with not having any cooperativity, namely a Hill coefficient 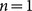. Thus, these biochemical measurements [47] strongly suggest that our results are not explained by single molecule cooperativity at the level of Sir2.

B) Cooperativity in the readout (Fig. 6B).

One could imagine a model in which the histone modification state of each nucleosome is independent, but the transcriptional readout depends on certain conditions being satisfied by the histone modification status of multiple nucleosomes. However, this scenario runs into two difficulties. First, cooperative transcription model needs to explain the 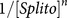 falloff, which will require a mechanism in which a 5–6 sites/nucleosomes need to be in the silenced state in order to stop transcription. This condition is somewhat implausible, both in models of silencing relying on hindrance of transcription initiation complex formation [48] and in those requiring blockage of processes like transcript extension subsequent to initiation. Secondly, under some conditions (like in *sas2* mutants), expression from a locus shows levels, that are in between a completely ‘on’ and a completely ‘off’ state [40], suggesting that expression is a graded function of the degree of silencing.

C) Polymerization/oozing (Fig. 6C).

This is the most traditional model of silencing [2] (sometimes derisively called the ‘railroad model’) where the feedback between the histone modification and SIR occupancy could easily lead to bistability and explain the hysteresis effect. This model naturally incorporates cooperation between multiple silencing complexes as the key ingredient for epigenetic stability. A quantitative model with nonlinear dynamics has been quite successful in explaining many features of the system [15], [49]. It is quite likely that the nonlinearity arises from multiple positive feedbacks involving more than one modification [16], [50].

In addition, this model naturally explains the nature of the bifurcation provided we allow for some silencing domain boundaries to be fixed dynamically, as shown in [15], [27]. Inhibiting Sir2p will not only shrink some silenced domains by altering the condition for balance at the dynamic boundary but also affect the two states, especially the quality of the silencing for the ‘off’ state. Ultimately, these effects lead to the merger of the states at the transition, without the need of any fine tuning [27]. Similar, but not the same, titration effect has been invoked to explain the intermediate expression level of *sas2* mutants [15], [40].

D) Looping and perinuclear localization (Fig. 6D).

Given that the telomeres are often found tethered to the nuclear periphery and that potential interactions between telomere and the hidden mating loci could bring those silenced loci to the same region [51], it is fair to ask whether silencing is just a passive readout of some feature related to that part of the nucleus, for example, higher than average abundance of silencing proteins. The simplest version of such a model, explaining silencing solely in terms of subnuclear localization of loci and the presence of a gradient of Sir proteins, would not be consistent with our data, unless the state of silencing has a strong feedback on subnuclear localization. Cooperative models, where partly silenced regions come together and further enhance silencing effect, would be discussed next. The simple model involving perinuclear localization, meanwhile, does not sit well with recent work [52] which shows that Sir3p overexpression leads to telomere clustering in the center of the nucleus, while not affecting silencing.

Let us consider a model that is very different from the polymerization/oozing model, but emphasizes subnuclear localization of a region as the key cause of epigenetic silencing. We might focus solely on regions with well-defined boundaries, like HMR [53] and probably quite a few telomeres [54], where one might imagine the whole region to be either in the silenced or in the active state. We have already argued that one needs strong positive feedback between subnuclear localization and the state of silencing to explain the observed hysteresis. However, such a model would predict a simple saddle-node bifurcation, in absence of any additional condition coming from dynamic adjustments of domain sizes, as discussed in the introduction. Such a prediction is contrary to our observation in the *ADE2* experiments, which suggests a supercritical pitchfork-like bifurcation.

E) Looping and clustering (Fig. 6E).

Chromatin looping and clustering of silenced region is observed in many systems, from SIR silencing in yeast to polycomb silencing in metazoans. One could propose that contact between many nucleosomes in a chromosomal unit is essential for stable silencing. The difference between this model and the linear polymerization model is that the number of potential ‘neighbors’ is much larger, and typically such a cluster-based model provides better epigenetic stability than one-dimensional models. Our observation of hysteresis effect is completely consistent with models involving interacting clusters. As long as these clusters could grow or shrink in response to drug dosage, in a manner analogous to the domain boundary shifts in the linear polymerization, we will be able to explain the merger of states at the threshold. As far as overall qualitative predictions, models C and E are very similar, apart from the issue of contiguity of silencing domains, something that is not being addressed by our experiments.

In summary, all these models do involve supramolecular cooperativity.

So far we have focused on the nature of cooperativity in silencing. Apart from this issue, there is an additional interesting feature in our observation. Notice that in traditional hysteresis experiments, crossing the saddle node bifurcation leads to a jump in the observed value. In contrast, in our data of survival fraction, and in the simulated SIR occupancy, we do not notice a pronounced jump. This feature may be indicative of significant negative feedback from titration effect: namely, reducing silenced regions make more silencers available for binding. Theoretical analysis shows how this brings the system close to the cusp in the bifurcation diagram [15], [27]. In particular, one can show that there are two different regimes, depending upon the overall abundance of silencers. In one regime, the system shows a jump, but in the other, the two states merge continuously. In the second regime, the system passes through the cusp, losing bistability, as one increases the amount of the Sir2p inhibitor [27]. Fig. 4A and 4B indicate the qualitative changes in the hysteresis curves in the presence of titration. Of course, we would need more careful experiments if we want to establish that the system indeed goes through a continuous transition.

One might point out that there are still many aspects of yeast silencing that are ill understood. In addition to Sir2p, yeast has four other NAD-dependent histone deacetylases, Hst1-4p (homologues of Sir2), some of which have locus specific roles in silencing [55]. Specifically, the double mutant *hst3 hst4* shows defect in telomeric silencing [55]. It appears that the action of splitomycin is primarily through inhibition of Sir2p, and then to some extent through reduction of Hst1p activity [47]. Such details would have to be addressed in an eventual detailed systems level model of yeast silencing. In the present study, however, we are just trying to establish that our observations fit the phenomenology of a sharp transition from bistability to monostability, as the inhibitor level is increased. Such a transition could happen even in a more complex model, with the *HST* genes playing a role.

Timescales of genetic and epigenetic changes play an important role in survival of an organism and are actively altered to suit demands of the environment [56]. Understanding key variables that affect these timescales, therefore, is crucial to the systems biology of epigenetics. For several model prokaryotic switches, where transcription factors play major role in the positive feedback, the question of epigenetic switching timescale have received thorough theoretical and computational treatment [57]–[59]. In contrast, mathematical analysis of chromatin-based epigenetics is just becoming a mainstream endeavor, as indicated in several recent reviews [60]–[62]. Recent advances through detailed genomic experiments investigating the SIR system [43], [54] as well as an explosion in quantitative modeling of the epigenetic chromatin state [63]–[67], often in systems beyond budding yeast, suggest that there is a lot more to be done. Our work, establishing the soundness of the description of the SIR system as a bistable switch, albeit with some additional twists related to dynamic equilibrium between silenced and active domains, paves the way to building a more complete systems level description of chromatin silencing in yeast.

## Materials and Methods

### Observation of hysteresis in 5-FOA survival rates

For the scheme of the hysteresis experiments with 5-FOA survival, see Fig. 1A and Fig. 1B. Yeast strain FEP100-10 [41], with an *URA3* gene located at telomere 11L, obtained from the Louis lab, was used in this study. Yeast culture was grown according to standard procedures [45]. Freshly grown YPD culture was diluted in two separate batches: one in regular YPD media and the other with YPD media containing 60 µM splitomycin. After 24 hrs of growth, cells from each batch were spun, washed twice with fresh YPD media, and then resuspended in 250 µl of YPD once more.

From the suspension containing the cells grown in absence of the drug, 10 µl was inoculated into 96-well 2.0 ml Deep Well plates with 1.6 to 1.8 ml of YPD media, with the 18 wells containing, respectively 0, 1, 2, 3, 4, 5, 6, 7, 8, 10, 12, 14, 16, 20, 30, 40, 50 and 60 µM of splitomycin. Similarly, 10 µl from the suspension from the cells grown in 60 µM splitomycin was inoculated into 18 other wells, with YPD medium containing exactly the same intermediate concentrations of the drug as in the previous case. Cells were allowed to grow for 24 hrs in these intermediate drug concentrations. Then, these cells were spun, washed twice with sterile water and resuspended in 100 µl volume.

From this culture, serial dilutions were made and plated on YPD plates and 5-FOA plates [33]. For cells grown at a particular intermediate concentration of splitomycin, the same level of drug was present in the corresponding 5-FOA plates. Colonies were counted after 2–4 days of growth at 30°C. The serial dilutions were adjusted so that one can get reliable counts of the colonies. Survival fraction 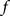 is defined as follows: 
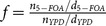
, where 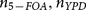 are the numbers of colonies in a particular type of plate, 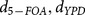 are the corresponding dilution factors which are always smaller than or equal to one. Percent errors in estimating the survival fractions were calculated based on expected variance of colony counts, separately for YPD and 5-FOA plates. The results are plotted in Fig. 2A.

### Computational modeling

In a related theoretical/computational work [46] we have proposed a minimal model of Sir silencing in yeast, identifying the key ingredients in the engineering design from experimentally known biological interactions. The model is minimal in demanding the least supra-molecular cooperativity of these interactions such that the system is capable of exhibiting epigenetic bistability.

We provide a brief summary of the model [46], which incorporates a “transcriptional feedback” in recruitment of active chromatin marks (H4K16 acetylation and H3K79 methylation), and “polymerization”-like feedback in recruitment and deacetylation activity of Sir proteins. Distinction is not made between the different Sir proteins in this minimal construction. The telomeric region of the chromosome is modeled as a lattice with nucleosomes as lattice sites. Each nucleosome can exist in multiple states, *unmodified (U)*, *acetylated (A), methylated (M), active (E),* and *silenced (S).* There are single site transitions (at site 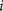) like 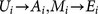 at the rate of Sas mediated basal acetylation. In addition, there are transitions that depend upon states at sites 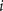 and 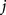 with rates depending on genomic separation between the sites. Some of the examples are as follows: 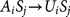 at the rate of cooperative Sir mediated deacetylation of neighbors of Sir occupied sites, ‘transcriptional feedback’ (potentially mediated by Dot1p activity [68], [69]) 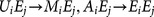 in neighbors of active sites, and finally cooperative Sir binding (Sir-polymerization) 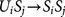.

We consider a single one-dimensional lattice of 200 sites to collectively represent all chromosomes. We impose boundary conditions such that the ‘telomere end’ of the lattice acts as a nucleation center of Sir binding while the other end is transcriptionally active. The net Sir supply is fixed such that if all Sir were lattice bound only 100 sites would be in state *S*. Sir titration effect is a result of this fixed Sir supply with the ambient Sir density determining the local availability of Sir. We use Gillespie algorithm for lattice simulations of the model reactions. All cell cycle-related perturbations are modeled as a constant loss of marks (i.e., a uniform rate of conversion of all other states to *unmodified*), which sets the scale for measuring all other reaction rates. We determine both the approach to *steady-state* and the steady-state behavior of the model in simulations by measuring the profile of modified states on the lattice.

In order to investigate hysteresis effects, we assume a ‘wild type’ choice of reaction rates to be a random choice for which the system exhibits robust bistable behavior, with well-defined and inheritable domains of Sir occupancy in the ‘telomeric end’ of the lattice. We assume that the effect of inhibitor is to inhibit the Sir activity by a factor 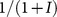, where *I* is the scaled concentration of inhibitor (*I = [Splito]/K_D_* in the experimental context). Firstly, we determine the steady state configurations at HIGH and LOW concentrations of inhibitors set to *I* = 4 and *I* = 0, respectively. In the experiments, this would correspond to growing the cells for several generations. Then we consider several such configurations as initial state and expose the state to the conditions of intermediate inhibitor level. *Short time* dynamics is ignored and *intermediate time* dynamics is reported. The *short time* dynamics is determined by the typical time it takes for a site to be selected in the simulation for its state to be updated. The *intermediate time* dynamics is determined by the supra-molecular interactions of the system, which is of interest. At *long times* all hysteretic memory-effects will be lost, especially in finite lattices. The *long-time* corresponds to probing the state of the cells after so many generations that no memory can be retained of what inhibitor concentration the cells were initially grown in. The intermediate-time behavior is plotted in Fig. 2B. The y-axis reports the Sir occupancy level in a scale of 0–1 of a locus (lattice sites 50-5 from the telomere). This Sir occupancy maps (perhaps nonlinearly) to the survival fraction of cells in a population; the more the *URA3* gene is suppressed the better the survival rate. The figure shows that the qualitative behavior of the experimental observations is reproduced in such a minimal model. We emphasize that realistic supra-molecular cooperativity (from experimentally known biochemistry) was an essential ingredient of this computational model.

### Studying the approach to bifurcation by pigmentation in *ADE2* silencing

Yeast strain UCC7366 (*MAT* **a** *ade2*Δ*::HisG his3*Δ*200 leu2*Δ*0 lys2*Δ*0 met15*Δ*0 trp1*Δ*63 ura3*Δ*0 ADE2-TEL-VR URA3-TEL-VIIL*) [70], a kind gift from Fred van Leeuwen’s lab, was used to study *ADE2* variegation near telomere. The strain was streaked out on YPD media. From YPD plate single colony was streaked out on to YC medium (as described in [71]) followed by inoculation into 4 ml of YC liquid medium.

Overnight grown culture was diluted one to 100 into fresh 2 ml YC medium, containing no splitomycin. Cultures were grown for 24 hours. The cells were pelleted, washed two times with 1 ml sterile water and finally re-suspended in 200 µl of water. From here, 10^−2^, 10^−4^ and 10^−6^ dilutions were made and 10 µl from 10^−4^ and 10^−6^ were plated onto 60×15 mm Petri dishes containing solidified 10 ml YC medium with various concentrations of splitomycin (spread on to the plates prior to use). After 3–4 days of growth, plates were kept over night in cold room and images were acquired using a 3^rd^ generation iPad 5 Megapixel iSight camera. The segmentation of the images to extract individual colonies was done using Image-Pro Premier v9.0 Image Analysis Demo software from Media Cybergenetics. The mean intensities for the three channels (RGB) for each colony were exported using the same software.

Further analysis of the channel intensities was done using Python and NumPy. In particular, Gaussian Mixture Model (GMM) from the module sklearn.mixture.GMM, courtesy scikit-learn 0.14 [72], was used to classify colonies in the regime that indicates two different epigenetic states. Intensities were normalized to the average intensity over all channels and colonies (to compensate for variation in overall illumination) coming from the same plate. For a plate with a particular concentration of splitomycin, each colony gave rise to a three dimensional vector, corresponding to three normalized channel intensities. We then fit the data to a single component three-dimensional Gaussian Model and to a two component three-dimensional GMM. We notice that the computational classification for the two-component GMM matches with the results from visual identification of ‘on’ or ‘off’ cells for concentrations of splitomycin up to a certain threshold. Beyond that threshold, the improvement from the two-component fit essentially captures the non-Gaussian nature of the unimodal distribution, rather than indicating the existence of two distinct groups of colonies. In this regime, we therefore use the single Gaussian fit.

The Gaussian models give us mean channel intensities for the ‘on’, ‘off’ and the unimodal groups. Rather than using a three-dimensional plot for these mean channel intensities for different concentrations, we plot the projection of these vectors along a particular direction that emphasizes the difference between on and off states. The direction was chosen to be the leading principal component of ‘on’-‘off’ normalized channel intensity vector differences for various splitomycin concentrations. The plots were done using matplotlib and pyplot.

## Supporting Information

S1 File **Analysis of single cell GFP data.** This file provides description of the experiments involving single cell gene expression at a silenced locus.
(DOCX)
