# Supplementary material for: Breaking an Epigenetic Chromatin Switch: Curious Features of Hysteresis in Saccharomyces cerevisiae Telomeric Silencing
Source: PLoS One. 2014 Dec 23;9(12):e113516. doi: 10.1371/journal.pone.0113516 (PMC4275178; doi:10.1371/journal.pone.0113516)
Supplement: S1 File — Analysis of single cell GFP data. This file provides description of the experiments involving single cell gene expression at a silenced locus. (DOCX) [file pone.0113516.s001.docx]

**Supporting Information File S1: Analysis of single cell GFP data**

**Measurement of Single Cell GFP fluorescence:**

Strain YDS1005(BM148), which contains a GFP reporter integrated at the HMR locus (Bitterman, Anderson et al. 2002), obtained from David Sinclair lab, was used for this study. Fresh cells from YPD media were inoculated into 4 ml of Synthetic Complete YC medium with or without 60 μM splitomycin, as described earlier in our previous protocol for *ADE2* color assay. Cells were grown for 2 to 3 days with change of fresh media. Then, the cells were spun, washed twice and resuspended in 100 to 200 μl of sterile water. From this cell suspension, 10 μl was inoculated into 4 ml of YC media containing, different concentrations of splitomycin. Cells were grown for 24 hours and images were acquired using Nikon TE 2000 S microscope equipped with CoolSNAP EZ camera. GFP intensities were extracted using FIJI (Schindelin, Arganda-Carreras et al. 2012), an open source image processing software based on ImageJ (Abràmoff, Magalhães et al. 2004). GFP intensities were extracted manually for about 10 fields each containing about 50 to 100 cells. The Region of Interest (ROI) was determined from in DIC images and were later overlaid on GFP images using ImageJ. These results were further processed using Python and histograms were plotted using Matplotlib and Pyplot.

**Comments on the observations:**

The results are summerized in Figure 1. As the concentration of the inhibitor splitomycin increases from 0 to 40 μM, first the expression increases and becomes heterogeneous. However as concentration goes even higher, there seems to be a reduction of intensity and reduction in heterogeneity.

We notice that the gene expression of single cells is extremely noisy, and strongly size-dependent (scatter-plots in the third column). One could argue that at an intermediate concentation (around 15 μM splitomycin), there are two populations of cells, as seen in the histogram of per pixel intensity. The histogram does not have two very well-defined separated peaks. This data is not unlike the results from Xu *et al* (Xu, Zawadzki et al. 2006). These two populations seem to come closer as concentration increases. We also see that the brightnees does not monotonically increase with increasing inhibitor concentration, something that tallies with our previous theoretical work (Dayarian and Sengupta 2013). However given how wide these distributions are and given the error in estimation, it is unlikely one could draw these conclusions with confidence.

Figure 1: The four columns provide information on the nature of single cell gene expression data. First column: concentration of splitomycin; second column: raw image of fluorescent cells from a typical field of view; third column: Scatter plot of background corrected intensity in arbitrary unit (y-axis) plotted against cell size in number of pixels (x-axis); fourth column: normalized histogram of per pixel background corrected intensity. The red and the green lines are guides to the eye for the high expression and the low expression population, obtained by two Gaussian mixture fit.

References:

Abràmoff, M. D., et al. (2004). "Image processing with ImageJ." Biophotonics international **11**(7): 36-43.

Bitterman, K. J., et al. (2002). "Inhibition of silencing and accelerated aging by nicotinamide, a putative negative regulator of yeast sir2 and human SIRT1." J Biol Chem **277**(47): 45099-45107.

Dayarian, A. and A. M. Sengupta (2013). "Titration and hysteresis in epigenetic chromatin silencing." Phys Biol **10**(3): 036005.

Schindelin, J., et al. (2012). "Fiji: an open-source platform for biological-image analysis." Nat Methods **9**(7): 676-682.

Xu, E. Y., et al. (2006). "Single-cell observations reveal intermediate transcriptional silencing states." Mol Cell **23**(2): 219-229.
